# Long-term kidney outcomes of semaglutide in obesity and cardiovascular disease in the SELECT trial

**DOI:** 10.1038/s41591-024-03015-5

**Published:** 2024-05-25

**Authors:** Helen M. Colhoun, Ildiko Lingvay, Paul M. Brown, John Deanfield, Kirstine Brown-Frandsen, Steven E. Kahn, Jorge Plutzky, Koichi Node, Alexander Parkhomenko, Lars Rydén, John P. H. Wilding, Johannes F. E. Mann, Katherine R. Tuttle, Thomas Idorn, Naveen Rathor, A. Michael Lincoff

**Affiliations:** 1 Institute of Genetics and Cancer, University of Edinburgh, Edinburgh, UK; 2 Department of Internal Medicine/Endocrinology, Peter O’Donnell Jr. School of Public Health, UT Southwestern Medical Center, Dallas, TX USA; 3 Novo Nordisk A/S, Søborg, Denmark; 4 Institute of Cardiovascular Sciences, University College London, London, UK; 5 Department of Medicine, VA Puget Sound Health Care System and University of Washington, Seattle, WA USA; 6 Division of Cardiovascular Medicine, Brigham and Women’s Hospital, Harvard Medical School, Boston, MA USA; 7 Department of Cardiovascular Medicine, Saga University, Saga, Japan; 8 National Scientific Centre named after M.D. Strazhesko, Kiev, Ukraine; 9 Department of Medicine Solna, Karolinska Institutet, Stockholm, Sweden; 10 Department of Cardiovascular and Metabolic Medicine, Institute of Life Course and Medical Sciences, University of Liverpool, Liverpool, UK; 11 KfH Kidney Centre, München, Germany, and Department of Nephrology and Hypertension, University Hospital, Friedrich-Alexander University, Erlangen, Germany; 12 Kidney Research Institute and Division of Nephrology, University of Washington, Seattle, WA USA; 13 Department of Cardiovascular Medicine, Cleveland Clinic Lerner College of Medicine, Case Western Reserve University, Cleveland, OH USA

**Keywords:** Cardiovascular diseases, Chronic kidney disease, Obesity, Randomized controlled trials

## Abstract

The SELECT trial previously reported a 20% reduction in major adverse cardiovascular events with semaglutide (*n* = 8,803) versus placebo (*n* = 8,801) in patients with overweight/obesity and established cardiovascular disease, without diabetes. In the present study, we examined the effect of once-weekly semaglutide 2.4 mg on kidney outcomes in the SELECT trial. The incidence of the pre-specified main composite kidney endpoint (death from kidney disease, initiation of chronic kidney replacement therapy, onset of persistent estimated glomerular filtration rate (eGFR) < 15 ml min^−1^ 1.73 m^−^^2^, persistent ≥50% reduction in eGFR or onset of persistent macroalbuminuria) was lower with semaglutide (1.8%) versus placebo (2.2%): hazard ratio (HR) = 0.78; 95% confidence interval (CI) 0.63, 0.96; *P* = 0.02. The treatment benefit at 104 weeks for eGFR was 0.75 ml min^−1^ 1.73 m^−^^2^ (95% CI 0.43, 1.06; *P* < 0.001) overall and 2.19 ml min^−1^ 1.73 m^−^^2^ (95% CI 1.00, 3.38; *P* < 0.001) in patients with baseline eGFR <60 ml min^−1^ 1.73 m^−^^2^. These results suggest a benefit of semaglutide on kidney outcomes in individuals with overweight/obesity, without diabetes.

ClinicalTrials.gov identifier: NCT03574597.

## Abstract (web summary)

In a pre-specified secondary analysis of the SELECT trial, once-weekly subcutaneous semaglutide 2.4 mg in patients with obesity was associated with a 22% reduction in the main 5-component kidney composite endpoint compared to patients on placebo.

## Main

Obesity is a risk factor for decline in glomerular filtration rate (GFR) and increased albuminuria^1,2^. Although part of this association is due to diabetes and associated cardiometabolic disturbances, even in individuals without diabetes, obesity increases the risk of an estimated glomerular filtration rate (eGFR) < 60 ml min^−1^ 1.73 m^−^^2^ (that is, stage 3 or worse chronic kidney disease (CKD)). For example, body mass index (BMI) was a predictive covariate in the CKD Prognosis Consortium risk equation for kidney failure or eGFR loss, based on 31 international cohorts in approximately 4.5 million individuals without diabetes^3^. Mendelian randomization studies found evidence that the association between overweight and obesity with CKD is causal. For example, for a 5-kg m^−^^2^ higher genetically predicted BMI, the odds of CKD were increased by 49%, with 30% not attributed to known mediators^4,5^.

Potential mechanisms underlying CKD in obesity included hyperfiltration, increased inflammation, oxidative stress, increased tubular sodium reabsorption and activation of the renin–angiotensin–aldosterone system^6,7^. Kidney pathological features associated with obesity included ectopic lipid accumulation, increased kidney sinus fat, hyperfiltration-related glomerular filtration barrier injury and obesity-related glomerulopathy (focal segmental glomerulosclerosis with or without glomerulomegaly)^2,8^. An important question is, therefore, how best to prevent or treat obesity-related CKD. An ideal therapy for obesity-related CKD would be one that reduces body weight and has direct effects on pathways involved in kidney injury. Glucagon-like peptide-1 (GLP-1) receptor agonists (GLP-1RAs) may have such direct kidney protective effects, with some evidence of altered expression of fibrosis-associated and inflammation-associated genes and downregulation of the receptor for advanced glycation end products^9–11^.

A meta-analysis of secondary analyses from large cardiovascular disease (CVD) outcome trials of GLP-1RAs in patients with type 2 diabetes and high cardiovascular risk showed an improvement in composite kidney outcome measures^12^. However, it remains unclear whether GLP-1RAs, such as semaglutide, are associated with beneficial effects on kidney function in individuals with overweight or obesity, without diabetes.

The SELECT trial demonstrated that once-weekly subcutaneous semaglutide 2.4 mg was associated with a 20% reduction in major adverse cardiovascular events (MACE) versus placebo in patients with pre-existing CVD and a BMI of ≥27 kg m^−^^2^, without diabetes^13^. In this pre-specified analysis of SELECT, we examined the effects of semaglutide on a range of kidney outcomes.

## Results

### Baseline characteristics

A total of 8,803 patients were randomized to semaglutide 2.4 mg and 8,801 to placebo. As shown in Supplementary Table 1, the baseline characteristics of patients in the SELECT trial were well balanced between treatment arms with respect to kidney function and albuminuria status as well as other characteristics. Of note, just over one-fifth of the trial population had either an eGFR <60 ml min^−1^ 1.73 m^−^^2^ or a urinary albumin-to-creatinine ratio (UACR) ≥ 30 mg g^−1^ at baseline. The median follow-up time was 182 weeks.

### Follow-up

As reported previously^13^, permanent premature discontinuation occurred in 2,351 patients (26.7%) in the semaglutide group and in 2,078 patients (23.6%) in the placebo group. Patients received the assigned trial product for 82.5% and 87.7% of the potential treatment time in the semaglutide and placebo arms, respectively. A total of 17,061 patients (96.9%) completed the trial (defined as having attended the final trial visit or died), and vital status was available for 17,495 patients (99.4%).

### Effect of semaglutide on the main kidney endpoint

**Fig. 1:**
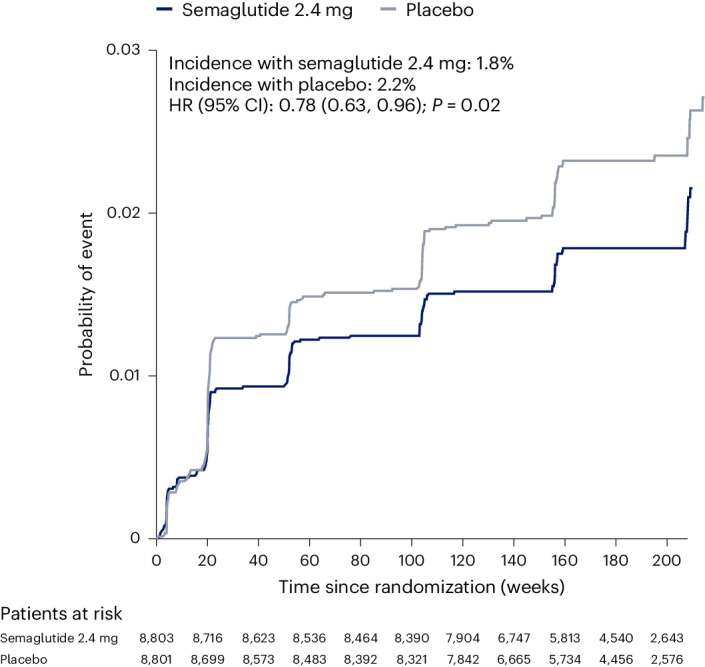
Time to first occurrence of the main 5-component kidney composite endpoint^a^. Data are the observed (that is, as measured) probability of patients experiencing their first occurrence of the main 5-component kidney composite endpoint during the in-trial period, analyzed using the Kaplan–Meier method, and the estimated HR, analyzed using a Cox regression model. Tied events were handled using the Exact method, if possible, or Efron’s method, if not. Numbers below the graph are the number of patients at risk. *P* values are two-sided and were not adjusted for multiplicity. ^a^ The main 5-component kidney composite endpoint included death from kidney causes, initiation of chronic kidney replacement therapy (dialysis or transplantation), onset of persistent eGFR <15 ml min^−1^ 1.73 m^−2^, persistent ≥50% reduction in eGFR compared to baseline or onset of persistent macroalbuminuria.

**Fig. 2:**
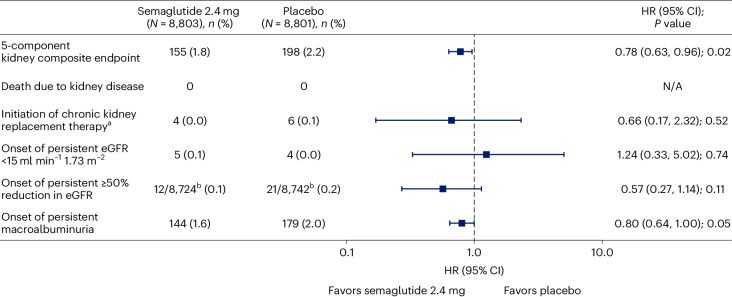
Effect of semaglutide 2.4 mg on the main 5-component kidney composite endpoint. Data are the observed (that is, as measured) *n* (%) of patients experiencing the first event that contributed to the main 5-component kidney composite endpoint from the in-trial observation period and the HR, and 95% CI was estimated using a Cox regression model. The symbols are the HRs, and the error bars are the 95% CIs. *P* values are two-sided and were not adjusted for multiplicity. ^a^ Dialysis or kidney transplantation. ^b^ Percent reduction is defined from baseline; the denominator is, therefore, reduced because of patients missing a baseline score. N/A, not applicable.

The pre-specified 5-component main kidney endpoint was the first specified death from kidney disease, initiation of chronic kidney replacement therapy (dialysis or transplantation), onset of persistent eGFR <15 ml min^−1^ 1.73 m^−^^2^, persistent ≥50% reduction in eGFR compared to baseline or onset of persistent macroalbuminuria. By the end of follow-up, 1.8% of patients randomized to semaglutide experienced this endpoint versus 2.2% of patients randomized to placebo (Fig. 1). Randomization to the semaglutide arm was associated with a hazard ratio (HR) of 0.78 (95% confidence interval (CI) 0.63, 0.96; *P* = 0.02) for the in-trial analysis. For the on-treatment analysis set, the HR was 0.75 (95% CI 0.59, 0.94; *P* = 0.01). Figure 2 shows the HR for the effect of treatment on the individual components of the composite. The effect on the main endpoint was driven by the treatment effect on onset of macroalbuminuria and persistent ≥50% reduction in eGFR, with the other components being sparse.

**Fig. 3:**
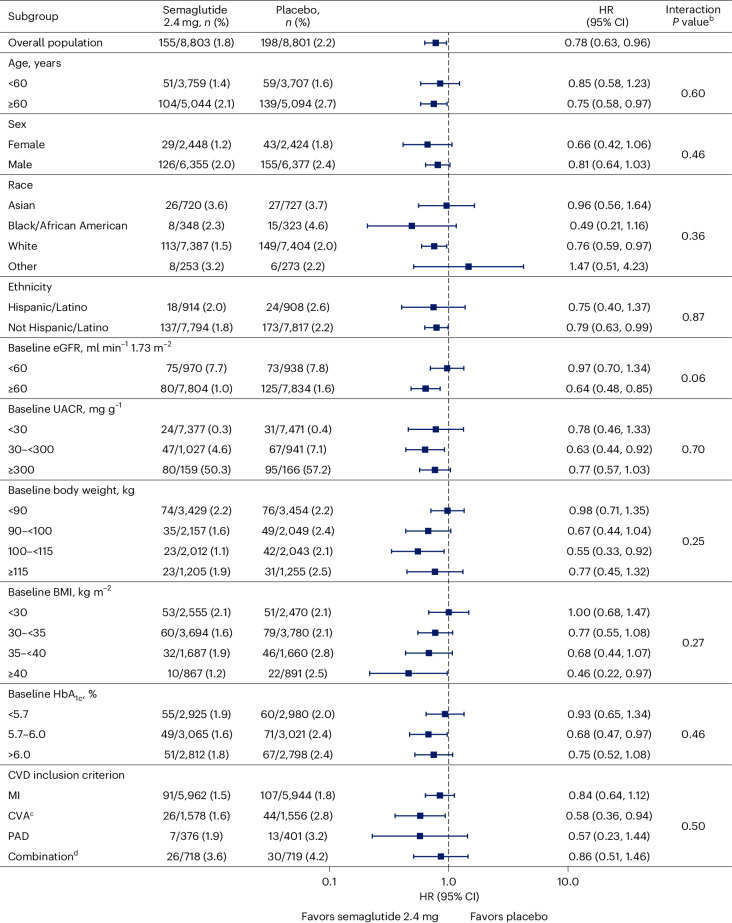
Effect of semaglutide 2.4 mg on the main 5-component kidney composite endpoint^a^ by subgroup. Data are the observed (that is, as measured) *n* (%) of patients experiencing their first occurrence of the main 5-component kidney composite endpoint from the in-trial observation period and the HR, and 95% CI was estimated using a Cox regression model, assessed according to patient baseline characteristics. The symbols are the HRs, and the error bars are the 95% CIs. ^a^ The main 5-component kidney composite endpoint included death from kidney causes, initiation of chronic kidney replacement therapy (dialysis or transplantation), onset of persistent eGFR <15 ml min^−1^ 1.73 m^−2^, persistent ≥50% reduction in eGFR compared to baseline or onset of persistent macroalbuminuria. ^b^ *P* value for the treatment difference by subgroup. Interaction *P* values were calculated using the score test. *P* values are two-sided and were not adjusted for multiplicity. ^c^ Ischemic or hemorrhagic stroke. ^d^ A combination of two or more events. CVA, cardiovascular accident; PAD, peripheral artery disease.

Figure 3 shows the effect of semaglutide on the main endpoint across subgroups defined by the selected baseline characteristics. No statistically significant interactions were observed in any subgroup with the treatment effect of semaglutide. In a post hoc analysis, the HR was 0.74 (95% CI 0.58, 0.94) in those 13,054 patients on an angiotensin-converting enzyme inhibitor (ACEi) or angiotensin receptor blockers (ARBs) and was 0.92 (95% CI 0.60, 1.42) in those 4,550 patients who were not on ACEi/ARB (*P* = 0.39 for the interaction). In total, 159 patients were in the semaglutide arm, and 166 patients were in the placebo arm, with a randomization UACR ≥ 300 mg g^−1^ (Fig. 3), of whom 80 and 95, respectively, experienced a main endpoint. The remainder of these patients did not develop persistent macroalbuminuria (or any of the other qualifying events for the main endpoint).

### Effect of semaglutide on eGFR at 104 weeks

**Fig. 4:**
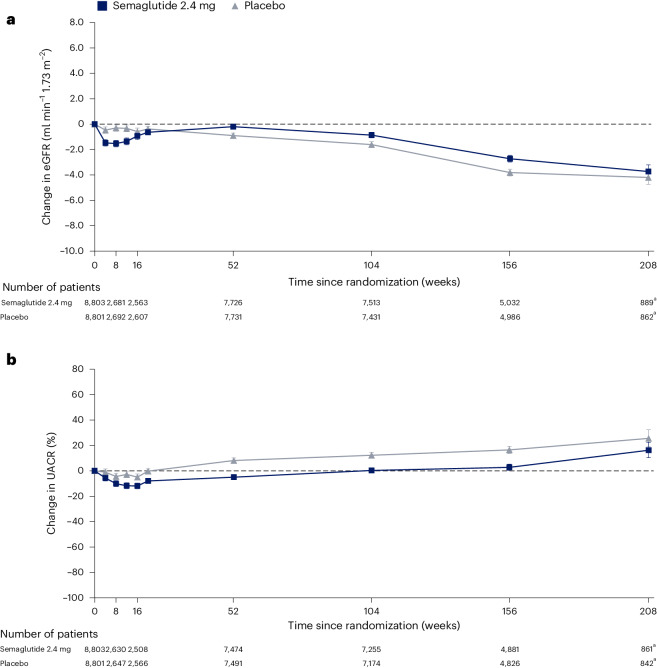
Effect of semaglutide 2.4 mg on changes in eGFR and UACR over time in the overall population. Data are estimated mean (CI) changes from the estimated baseline value in eGFR (**a**) and UACR (**b**), analyzed using an MMRM. The change in UACR was analyzed as the estimated mean ratio to baseline; for ease of interpretation; these ratios were converted to relative percentage changes from baseline using the formula (estimated ratio − 1) × 100. Numbers below the graphs are the number of patients contributing to the analysis. ^a^ Given gradual entry to the trial across the enrolment period and variable follow-up duration, data at 156 weeks and 208 weeks are sparser compared to previous timepoints.

At the pre-specified timepoint of 104 weeks, the mixed model for repeated measures (MMRM) estimated that eGFR had declined less in the semaglutide arm (−0.86 ml min^−1^ 1.73 m^−^^2^) versus the placebo arm (−1.61 ml min^−1^ 1.73 m^−^^2^), giving a net treatment benefit of 0.75 ml min^−1^ 1.73 m^−^^2^ (95% CI 0.43, 1.06; *P* < 0.001) for the overall population (Table 1 and Fig. 4a).

**Table 1:** Effect of semaglutide 2.4 mg on continuous kidney endpoints

Outcome	Semaglutide 2.4 mg (*N* = 8,803)	Placebo (*N* = 8,801)	ETD (95% CI); *P* value
Change in eGFR at week 104, mL min^−1^ 1.73 m^−2^	−0.86	−1.61	0.75 (0.43, 1.06); *P* < 0.001
By baseline eGFR			
<60 ml min^−1^ m^−2^	5.28	3.09	2.19 (1.00, 3.38); *P* < 0.001
≥60 ml min^−1^ m^−2^	−1.62	−2.20	0.57 (0.26, 0.89); *P* < 0.001
Total eGFR slope, ml min^−1^ m^−2^ per year	−0.78	−1.17	0.39 (0.30, 0.48); *P* < 0.001
Chronic eGFR slope, ml min^−1^ m^−2^ per year	−0.98	−1.28	0.29 (0.18, 0.40); *P* < 0.001
Acute eGFR slope, ml min^−1^ m^−2^ per year	−2.41	−1.08	−1.33 (−2.68, 0.02); *P* = 0.05
Change in UACR at week 104, log-transformed, %	0.3	12.3	−10.7 (−13.2, −8.2); *P* < 0.001
By baseline UACR			
<30 mg g^−1^	14.2	24.3	−8.1 (−10.6, −5.6); *P* < 0.001
30 to <300 mg g^−1^	−53.4	−36.0	−27.2 (−35.3, −18.1); *P* < 0.001
≥300 mg g^−1^	−75.0	−63.5	−31.4 (−54.9, 4.3); *P* = 0.08
By baseline eGFR			
<60 ml min^−1^ m^−2^	4.0	19.7	−13.1 (−22.1, −3.1); *P* = 0.01
≥60 ml min^−1^ m^−2^	−0.3	11.9	−10.9 (−13.5, −8.4); *P* < 0.001

For changes in eGFR and UACR, data are the estimated mean changes from the estimated baseline value to week 104 and ETD, analyzed using MMRM. Changes in UACR were analyzed as estimated mean ratio to baseline at week 104 and estimated treatment ratio; for ease of interpretation, these ratios were converted to relative percentage changes from baseline and relative percentage treatment differences using the formula (estimated ratio − 1) × 100. The eGFR slope (that is, the annualized rate of change in eGFR) and ETD were analyzed using a linear random regression model. The chronic period included data from week 20 to end of trial (that is, any data before week 20 were excluded). Total refers to the eGFR change from baseline to end of trial. The acute period included data from baseline to week 16 and was evaluable only in European patients in whom there were additional early measures. *P* values (from top to bottom of the table) are 2.6895 × 10^−6^, 0.0003, 0.0004, 1.9939 × 10^−16^, 1.3273 × 10^−7^, 0.0535, 3.5231 × 10^−15^, 1.2913 × 10^−9^, 1.4054 × 10^−7^, 0.0777, 0.0119 and 2.3245 × 10^−15^. *P* values are two-sided using a *t*-test in the models and were not adjusted for multiplicity. ETD, estimated treatment difference.

**Fig. 5:**
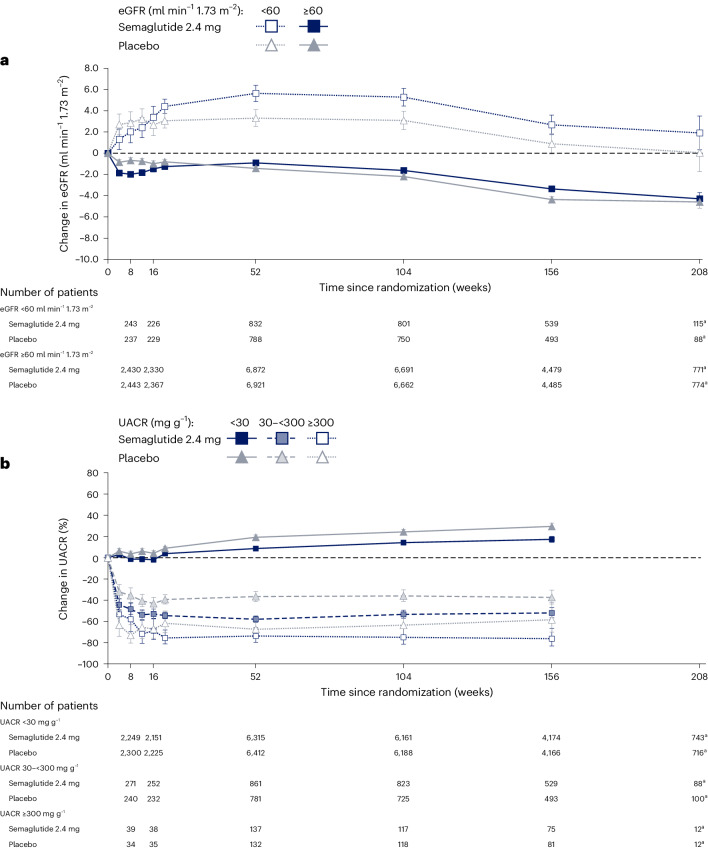
Effect of semaglutide 2.4 mg on changes in eGFR and UACR over time by subgroup. Data are estimated mean (CI) changes from the estimated baseline value in eGFR (**a**) and UACR (**b**), analyzed using an MMRM. The change in UACR was analyzed as the estimated mean ratio to baseline; for ease of interpretation, these ratios were converted to relative percentage changes from baseline using the formula (estimated ratio − 1) × 100. Darker lines are used for the larger subgroups. Numbers below the graphs are the number of patients contributing to the analysis. Changes in UACR by baseline UACR subgroup at 208 weeks are not presented because of small patient numbers. ^a^ Given gradual entry to the trial across the enrollment period and variable follow-up duration, data at 156 weeks and 208 weeks are sparser compared to previous timepoints.

**Extended Data Fig. 1:**
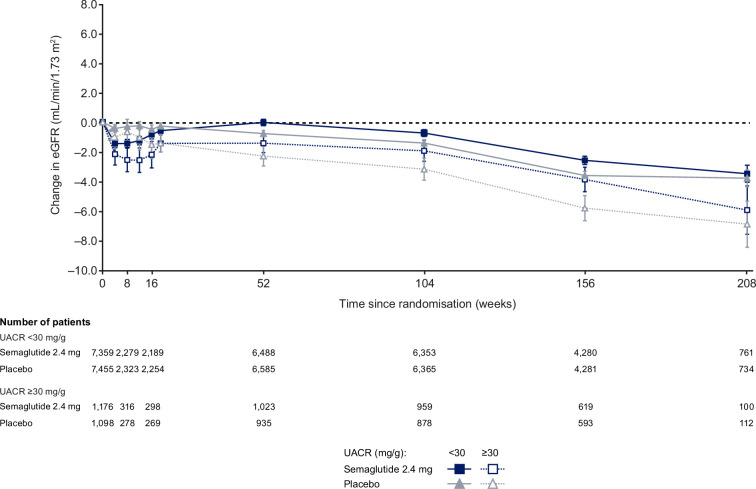
Effect of semaglutide 2.4 mg on changes in eGFR over time by baseline UACR. Data are estimated mean (CI) changes from the estimated baseline value in eGFR, analysed using a mixed model for repeated measurements. Darker lines are used for the larger subgroups. CI, confidence interval; eGFR, estimated glomerular filtration rate; UACR, urinary albumin-to-creatinine ratio.

In patients with baseline eGFR ≥60 ml min^−1^ 1.73 m^−^^2^ at 104 weeks, eGFR had declined less in the semaglutide arm versus the placebo arm, with an estimated treatment difference of 0.57 ml min^−1^ 1.73 m^−^^2^ (95% CI 0.26, 0.89; *P* < 0.001; Table 1 and Fig. 5a). In the subgroup with eGFR <60 ml min^−1^ 1.73 m^−^^2^ at baseline, there was a rise in eGFR that was greater with semaglutide (5.28 ml min^−1^ 1.73 m^−^^2^) versus placebo (3.09 ml min^−1^ 1.73 m^−^^2^) at 104 weeks (treatment difference: 2.19 ml min^−1^ 1.73 m^−^^2^; 95% CI 1.00, 3.38; *P* < 0.001; Table 1 and Fig. 5a). The changes in eGFR in each treatment arm in patients with and without albuminuria at baseline are shown in Extended Data Fig. 1.

### Effect of semaglutide on total and chronic eGFR slope

The total eGFR slope analysis (Table 1) showed that the fall in eGFR expressed per year across the entire trial duration was −0.78 ml min^−1^ 1.73 m^−^^2^ per year in patients randomized to semaglutide compared to −1.17 ml min^−1^ 1.73 m^−^^2^ per year in patients randomized to placebo, giving a 0.39 ml min^−1^ 1.73 m^−^^2^ per year lower slope in patients receiving semaglutide versus placebo (95% CI 0.30, 0.48; *P* < 0.001). The chronic slope estimate (that is, fall in eGFR expressed per year from 20 weeks onwards) showed a similar treatment effect of a 0.29 ml min^−1^ 1.73 m^−^^2^ per year lower slope in patients randomized to semaglutide.

### Effect of semaglutide on acute eGFR to week 20

As shown in Fig. 4a, in the patients from European centers (34.1% of all patients) in whom eGFR was measured at additional timepoints up to 16 weeks, there was an initial decline in eGFR that was more pronounced in the semaglutide arm, reaching its nadir at 8 weeks. The acute slope (that is, the annualized difference in eGFR between treatment arms in this short period of 16 weeks) was a −1.33 ml min^−1^ 1.73 m^−^^2^ per year greater decline in patients randomized to semaglutide. By week 20, eGFR was similar in both treatment arms overall.

### Correlation and mediation between eGFR change and changes in body weight, blood pressure and glycated hemoglobin

This study had insufficient power for a formal mediation analysis of the main composite kidney endpoint. Exploratory analyses indicated that there was little correlation between the within-person change in eGFR and the within-person changes in body weight, systolic blood pressure or glycated hemoglobin (HbA_1c_) in the semaglutide or placebo arms (Supplementary Table 2). However, a mediation analysis suggested that 81% (95% CI 41.30, 120) of the change in eGFR was attributable to change in body weight with considerable imprecision in the estimate.

### Effect of semaglutide on UACR

The MMRM-estimated changes in UACR over time in the overall population and by UACR subgroup are shown in Fig. 4b and summarized in Table 1. At 104 weeks, the UACR was estimated to have risen less in terms of percentage change from baseline in the semaglutide versus placebo arm, giving a net treatment benefit of −10.7% (95% CI −13.2, −8.2; *P* < 0.001) for the overall population.

**Extended Data Fig. 2:**
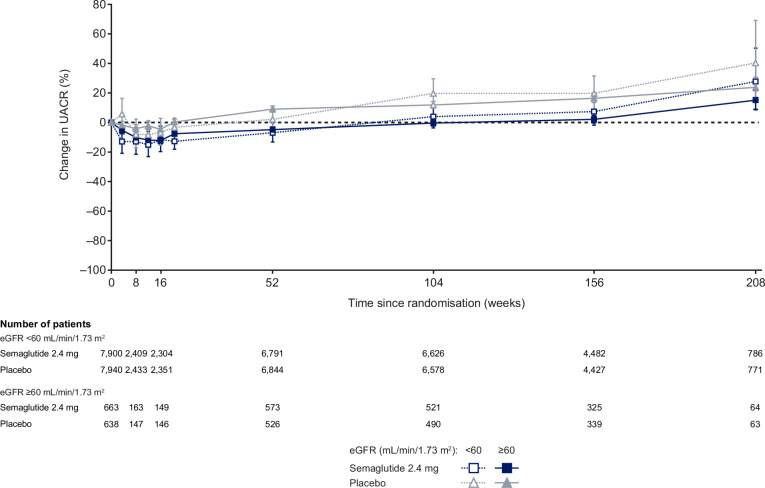
Effect of semaglutide 2.4 mg on changes in UACR over time by baseline eGFR. Data are estimated mean (CI) changes from the estimated baseline value in UACR, analysed using a mixed model for repeated measurements. The change in UACR was analysed as the estimated mean ratio to baseline; for ease of interpretation, these ratios have been converted to relative percentage changes from baseline using the formula (estimated ratio − 1) × 100. Darker lines are used for the larger subgroups. Numbers below the graphs are the number of patients contributing to the analysis. CI, confidence interval; eGFR, estimated glomerular filtration rate; UACR, urinary albumin-to-creatinine ratio.

When assessed by UACR subgroup (Fig. 5b), the pattern for patients with UACR at randomization <30 mg g^−1^ was similar to the overall study population (treatment difference: −8.1%; 95% CI −10.6, −5.6; *P* < 0.001). In patients with UACR ≥ 30 mg g^−1^ (Table 1), there was a fall in UACR that was greater in patients randomized to semaglutide versus placebo, with treatment differences of −27.2% (95% CI −35.3, −18.1; *P* < 0.001) for patients with UACR ≥ 30 mg g^−1^ to <300 mg g^−1^ and −31.4% (95% CI −54.9, 4.3; *P* = 0.08) for patients with UACR ≥ 300 mg g^−1^. A similar trajectory of UACR change over time and treatment effect was seen when stratified by baseline eGFR (Extended Data Fig. 2).

### Effect of semaglutide on other pre-specified kidney endpoints

Supplementary Table 3 summarizes the effect of semaglutide versus placebo on the other composite kidney endpoints and time to specific eGFR fall thresholds. For a 5-component endpoint that differed from the main 5-component endpoint in excluding incident macroalbuminuria but including death from cardiovascular causes, the HR was 0.82 (95% CI 0.69, 0.97; *P* = 0.02). Due to a small number of events, treatment effects on the additional endpoints, including two further composite endpoints that excluded macroalbuminuria, were not significant, even though the point estimates were consistent with HRs all below 1.

### Adverse events by baseline eGFR

The adverse events occurring in the two treatment arms were reported previously^13^. In brief, there were more adverse events leading to permanent discontinuation in patients receiving semaglutide (16.6% of patients randomized) versus placebo (8.2%; *P* < 0.001), mostly comprising gastrointestinal effects. Supplementary Table 4 shows that, within both treatment arms, such discontinuations were more common in patients with baseline eGFR <60 ml min^−1^ 1.73 m^−^^2^, with gastrointestinal effects predominating. There was no excess of acute kidney failure in patients randomized to semaglutide, regardless of baseline eGFR.

## Discussion

In this pre-specified analysis of the SELECT trial, we found that allocation to once-weekly subcutaneous semaglutide 2.4 mg in patients living with overweight or obesity was associated with a 22% reduction in the main 5-component kidney composite endpoint. The treatment effect on this event-based endpoint was driven by reduced incidence of macroalbuminuria and onset of persistent ≥50% reduction in eGFR.

We confirmed consistent direction of treatment effects for other pre-specified composites, including a significant 18% reduction in a kidney composite endpoint that comprised death from kidney causes, initiation of chronic kidney replacement therapy, onset of persistent eGFR <15 ml min^−1^ 1.73 m^−^^2^, persistent ≥50% reduction in eGFR or death from CVD causes. This composite is used as a primary endpoint in dedicated kidney disease outcome trials, including the FLOW trial (NCT03819153) of semaglutide in individuals with type 2 diabetes and CKD. FLOW was stopped early as pre-specified efficacy criteria were met in an interim analysis and is due to report in 2024 (ref. ^14^).

Analysis of the continuous eGFR endpoint at 104 weeks showed a significantly lesser decrease in eGFR in patients receiving semaglutide versus placebo. Although the effect was modest in the total population, in patients with eGFR <60 ml min^−1^ 1.73 m^−^^2^ at baseline, there was a rise in eGFR that was greater with semaglutide, giving a treatment benefit of 2.19 ml min^−1^ 1.73 m^−^^2^ at 104 weeks. Because a rise was observed in both arms in patients with eGFR <60 ml min^−1^ 1.73 m^−^^2^ at baseline, the rise may partly reflect regression to the mean. Such regression is to be expected when a threshold value of a highly variable measurement such as eGFR is used to define categories. Whether the treatment benefit reflects a net increase in eGFR in the semaglutide group or prevention of a fall is uncertain. Regardless of which of these is occurring, the treatment benefit is clear and substantial. This is a good example of why comparator groups are essential in trials.

The annualized treatment effect on eGFR across the total study period—that is, the slope—was a benefit of 0.39 ml min^−1^ 1.73 m^−^^2^ per year. To put this into context, it was previously estimated from a meta-analysis of 47 trials in over 60,000 individuals (one-third without diabetes) that a benefit of 0.72 ml min^−1^ 1.73 m^−^^2^ per year mean difference in total GFR slope over 2 years confers a 97.5% probability of clinical benefit (HR ~ 0.7) over the longer term in the endpoints of doubling of serum creatinine, incidence of eGFR <15 ml min^−1^ 1.73 m^−^^2^ or end-stage kidney disease^15,16^.

There was also a significant benefit of semaglutide on UACR with a net benefit of 10.7%. In patients with UACR ≥ 30 mg g^−1^ and ≥300 mg g^−1^ at randomization, the UACR fell substantially in both treatment arms after randomization, consistent with regression to the mean. However, this fall was greater for the semaglutide arm than the placebo arm, giving a net 27% and 31% treatment benefit in patients with randomization UACR ≥ 30 mg g^−1^ and ≥300 mg g^−1^, respectively. Whether this treatment benefit reflects an underlying reduction in UACR or the prevention of a rise in the absence of treatment cannot be differentiated. To put these treatment benefits into context, a meta-analysis of trials showed that a 30% reduction in estimated UACR predicts a 27% lower hazard risk of the clinical endpoint of doubling of serum creatinine, incidence of eGFR <15 ml min^−1^ 1.73 m^−^^2^ or end-stage kidney disease^17^.

These data from individuals with overweight or obesity and high cardiovascular risk are important as they constitute the first evidence to suggest that GLP-1RAs, and, specifically, semaglutide, could have beneficial effects on the kidney in the absence of diabetes. The finding of beneficial effects of semaglutide on kidney outcomes in this population is consistent with previous secondary analyses from trials of semaglutide and other GLP-1RAs^12,18–27^. Large CVD outcome trials of GLP-1RAs in individuals with type 2 diabetes and high cardiovascular risk showed an improvement in composite kidney outcome measures^12,18,19,21–27^. A meta-analysis of the ELIXA (lixisenatide), LEADER (liraglutide), SUSTAIN-6 (semaglutide), EXSCEL (exenatide), REWIND (dulaglutide) and AMPLITUDE-O (efpeglenatide) trials reported a reduction in the composite kidney outcome of development of macroalbuminuria, substantially worsening kidney function, kidney replacement therapy or kidney death by 21% (*P* < 0.001)^12^. Trial-specific effect estimates ranged from a 12% reduction in EXSCEL to a 36% reduction in SUSTAIN-6 (refs. ^12,18,19,21–27^). A secondary analysis of an open-label trial of the dual agonist tirzepatide in individuals with diabetes also suggested potential benefit on kidney outcomes^28^. Our findings are consistent with a previous exploratory analysis of the STEP 2 trial of semaglutide in individuals (*n* = 1,210) with overweight and obesity, which found significant reductions in UACR with semaglutide 1.0 mg and 2.4 mg weekly, with differences versus placebo of −28.0% and −32.9%, respectively^20^, resulting from an increase in UACR in the placebo group and a decrease in the semaglutide group^14^. The mechanism underpinning the effect of semaglutide on kidney outcomes is unclear. Of note, there was little correlation between the within-individual change in eGFR and change in body weight, HbA_1c_ or systolic blood pressure. However, a mediation analysis suggested that 81% of the change at week 104 could be mediated by body weight change. Such data could also arise if the mediation were through factors highly correlated with body weight change. Thus, weight loss likely contributes to some degree, and other interventions that result in large losses in weight, including bariatric surgery, have resulted in improvements in eGFR and albuminuria. For example, a meta-analysis of 19 studies (some with no comparator) reported an improvement of 12 ml min^−1^ 1.73 m^−^^2^ in eGFR postoperatively^29^. A previous mediation analysis in the LEADER and SUSTAIN-6 trials in individuals with type 2 diabetes suggested that reductions in HbA_1c_ and systolic blood pressure may moderately mediate kidney benefits of liraglutide and semaglutide^30^. Body weight was also a mediator in LEADER. A secondary mediation analysis of the STEP 2 trial of semaglutide in individuals with diabetes suggested that body weight change also contributed to change in UACR^20^. There is also some evidence of direct effects of GLP-1RAs on the kidney, including glomerular hemodynamic effects, reductions in inflammation and oxidative stress, increased natriuresis and diuresis and altered expression of the receptor for advanced glycation end products^9,11,20,31,32^. We do not have measurements of these mechanisms in SELECT.

An important question is whether apparent changes in Chronic Kidney Disease Epidemiology Collaboration (CKD-EPI) eGFR using serum creatinine with changing weight in a population with overweight and obesity reflects underlying changes in kidney function. This is a complex issue; there is the question of whether directly measured glomerular filtration rate (mGFR) is a better measure of underlying kidney function with or without the conventional indexing to body surface area (BSA) of 1.73 m^2^. The argument for indexing is that individuals with larger body size have higher GFR by virtue of body mass alone, but it has also been argued that such indexing may introduce error at extreme BMIs. Error at extreme BMIs may occur because both muscle and fat mass are increased in obesity, and serum creatinine increases with increased muscle mass. When weight loss occurs, both lean muscle mass and fat mass are reduced, and this loss of muscle mass can cause an artifactual rise in the indexed eGFR^33^. A related issue is that the CKD-EPI equation we used was trained to predict mGFR indexed or standardized to BSA rather than unindexed mGFR. The distribution of BMI in the population in which it was trained was less extreme than that in SELECT. Thus, whether the CKD-EPI creatinine-based eGFR is as reasonable a proxy for indexed mGFR in the settings of obesity and large changes in body weight is unclear, and studies of this question are conflicting^34–37^. A recent editorial advocated for the use of deindexed eGFR in the setting of weight loss^33,38^, but this is not a recommendation in the recent guidelines from the Kidney Disease Improving Global Outcomes (KDIGO) organization^39^. That said, a caveat of any reduction in estimated GFR based on creatinine in a trial where treatment is associated with weight (and, thereby, muscle mass) loss must consider contributions from both creatinine production and improved kidney filtration. In SELECT, there is coexistent improvement in UACR and eGFR, a finding that supports a benefit on kidney function.

An alternative to creatinine-based eGFR is the use of cystatin C in combination with creatinine. Cystatin C is not affected by muscle mass, but the difficulty in the setting of obesity and weight loss is that it may be affected by fat mass^40,41^. However, its use is now advocated, as this has been shown to improve the predictive performance of eGFR for measured GFR^42^ in the general population and in the context of weight loss^43^. Use of the creatinine and cystatin C–based estimated glomerular filtration rate (eGFRcr-cys) was highlighted in the recent KDIGO guidelines^39^. We have not measured cystatin C as yet in SELECT, but this would provide further clarity on the eGFR effects^43^. In a recent post hoc analysis, the effect of tirzepatide on eGFR was similar regardless of whether creatinine or cystatin C was used^44^.

In the European patients, where additional supplementary measures were done up to week 16, we noted an early fall in eGFR that was greater with semaglutide versus placebo, but, as shown in Fig. 4a, by 20 weeks there was no difference between treatment arms, and, by 52 weeks, eGFR was higher in the semaglutide versus placebo group. Reassuringly, this acute drop in eGFR associated with treatments that have longer-term benefit on eGFR was noted for sodium–glucose co-transporter-2 inhibitors, for renin–angiotensin–system inhibitors and for tirzepatide^28,45–47^. The cause for this transient worsening in eGFR is unclear, but alterations in glomerular pressure or natriuresis associated with GLP-1RA may contribute. Part of the initial drop in eGFR may be a regression to the mean effect given the recognized day-to-day variability in eGFR measures and given that it was observed in both arms. Reassuringly, there was a fall in UACR that was greater in the semaglutide versus placebo arm during these weeks. Two ongoing mechanistic studies, REMODEL (NCT04865770) in type 2 diabetes and SMART (NCT04889183) in individuals with overweight or obesity, will allow greater insight into local effects on the kidney that might underpin the overall treatment benefit and, in the acute phase, a greater fall in eGFR with semaglutide^48^.

Strengths of the present study are the large sample size, the randomized design and the duration of follow-up, allowing a demonstration that the beneficial effect on eGFR and UACR is maintained. This work is novel in demonstrating a potential beneficial kidney effect of semaglutide in the absence of diabetes and is the first demonstration, to our knowledge, of potential benefit in individuals with overweight or obesity. Although the effect of semaglutide on kidney outcomes was a secondary analysis of SELECT, with the primary endpoint being MACE, the analysis presented here was pre-specified.

This study also has a few limitations. Unlike most kidney endpoint trials, we did not selectively include patients at most risk of kidney disease progression. As a consequence, the number of kidney event endpoints and the power to examine effects on endpoints and detect subgroup interactions are limited. The allocated study drug was in addition to usual care, and not all participants were using guideline-directed treatments, namely ACE inhibitors or ARBs, for CKD progression. Another limitation is that we did not measure cystatin C–based eGFR to separate eGFR changes due to muscle mass or filtration. Furthermore, the number of events of the main composite endpoint was too low to support a formal mediation analysis of this endpoint. Although we include an estimate of the possible mediation in eGFR treatment effect by weight change, such estimates should be treated as suggestive, not definitive.

In conclusion, individuals with overweight and obesity constitute a high-risk population for incidence and progression of diabetes and its complications. Prevention of CKD is an important cornerstone of their clinical management. The 22% reduction in our pre-specified main kidney endpoint; the treatment benefit on eGFR, especially in patients with eGFR <60 ml min^−1^ 1.73 m^−^^2^; and the clinically relevant reductions in UACR in patients with albuminuria at baseline suggest a beneficial kidney effect of once-weekly subcutaneous semaglutide 2.4 mg in this at-risk population.

## Methods

### Trial design and patients

The design, baseline characteristics and primary results of SELECT were reported previously^13,49,50^. In brief, SELECT was a randomized, double-blind, placebo-controlled, event-driven trial, comparing semaglutide 2.4 mg with placebo added to standard of care for prevention of MACE in 17,604 individuals with established CVD and overweight/obesity, without type 2 diabetes.

Eligible patients were aged ≥45 years with a BMI of ≥27 kg m^−^^2^ and established CVD, defined as one or more of the following: prior myocardial infarction (MI), prior ischemic or hemorrhagic stroke or symptomatic peripheral artery disease. Exclusion criteria included a history of type 1 or type 2 diabetes; HbA_1c_ ≥ 6.5% (48 mmol mol^−1^); presence of end-stage kidney disease or need for dialysis; MI, stroke, hospitalization for unstable angina pectoris or a transient ischemic attack within 60 d before screening; or New York Heart Association Class IV heart failure. Patients were randomized 1:1 to receive escalating doses of once-weekly subcutaneous semaglutide over 16 weeks, to a target dose of 2.4 mg, or placebo.

### Ethics

The SELECT trial was conducted at 804 sites in 41 countries and was approved by the relevant institutional review board and/or ethics committee for each center. All patients provided written informed consent.

### Outcomes

The 5-component main kidney endpoint pre-specified in the protocol was the first of death from kidney disease, initiation of chronic kidney replacement therapy (dialysis or transplantation), onset of persistent eGFR <15 ml min^−1^ 1.73 m^−^^2^, persistent ≥50% reduction in eGFR compared to baseline or onset of persistent macroalbuminuria. Persistent was defined as two or more new measures at least 4 weeks apart. Patients with a UACR ≥ 300 mg g^−1^ at randomization were considered at risk of incident macroalbuminuria during follow-up. A complete list of CKD outcomes assessed is presented in Supplementary Table 5. These outcomes included other composite kidney endpoints, time to reaching various thresholds of percentage eGFR decline (30%, 40%, 50% and 57%), the annualized rate of change in eGFR (eGFR slope), change in eGFR from baseline to week 104 and change in UACR from baseline to week 104. Treatment effects on outcomes were further assessed by baseline eGFR and UACR status. Safety was assessed as the number of treatment-emergent adverse events.

Laboratory tests were performed by a central laboratory. Blood samples were collected at screening, at weeks 20, 52, 104, 156 and 208, and at the end of treatment. eGFR values were calculated centrally based on serum creatinine using the Chronic Kidney Disease Epidemiology Collaboration (CKD-EPI) 2009 creatinine equation^51^, with sensitivity analyses using the CKD-EPI 2021 equation without race conducted for confirmation of findings (data not shown)^52^. A confirmatory test was required at the onset of either a ≥50% reduction in eGFR or eGFR <15 ml min^−1^ 1.73 m^−^^2^. UACR was calculated based on single urine samples (preferably morning) that were collected at randomization, at weeks 20, 52, 104, 156 and 208 and at the end of treatment. A confirmatory test was performed at the onset of macroalbuminuria (>300 mg g^−1^). As an additional safety measure, all patients in Europe had additional laboratory data collection at weeks 4, 8, 12 and 16, with creatinine and UACR tests performed at the central laboratory, and the CKD-EPI 2021 equation used for eGFR.

All components of the main kidney 5-composite endpoint were adjudicated by an independent expert committee blinded to treatment status. Acute kidney failure events (also adjudicated) were those that involved an abrupt decrease in kidney function (for example, one of ≥0.3 mg dl^−1^ (≥26.5 μmol l^−1^) increase in serum creatinine within 48 h, ≥1.5 times increase in serum creatinine within 7 d or urine volume <0.5 ml kg^−1^ h^−1^ for 6 h).

Although the original study protocol used the terms ‘5-component nephropathy composite’ and ‘acute renal failure’, here, we rephrased these as ‘5-component kidney composite’ and ‘acute kidney failure’, in line with recent nomenclature guidance introduced since the writing of the protocol^53^.

### Statistical analysis

Baseline characteristics were summarized with means and standard deviations, medians and interquartile ranges, geometric means and coefficients of variation or counts and percentages, as appropriate. Efficacy analyses were performed on two sets: the full analysis set, defined as all unique randomized patients grouped according to the treatment assigned at randomization, thereby following the intent-to-treat principle, and an on-treatment analysis, defined as all exposed patients while they were receiving treatment (that is, had received at least one dose in the previous 35 d), grouped according to the treatment that they received. Analyses were based on the in-trial period, which was the time from randomization to the end-of-trial visit, death, withdrawal of consent or last contact with a trial site, whichever occurred first. Where applicable, analyses were conducted according to subgroups at baseline, based on pre-defined demographic, kidney, body weight, glycemic and CVD comorbidity characteristics. Significance levels were set at 5% (two-sided) with no adjustment for multiplicity. A sensitivity analysis using only data from the on-treatment period, which was the time from the first dose of trial product to 35 d after the last dose, excluding any temporary interruptions in taking trial product, was conducted. SAS analysis software (version 9) was used throughout.

Adverse events were summarized with counts and percentages. Safety analyses were performed on the safety analysis set, which included all randomized patients exposed to at least one dose of trial product, stratified by baseline eGFR.

#### Time-to-event analyses

HRs for event-based outcomes comparing semaglutide 2.4 mg versus placebo were estimated from a pre-specified Cox proportional hazards model with treatment group (semaglutide and placebo) as a binary variable, presented with two-sided 95% CIs and two-sided *P* values. Tied events were handled using the Exact method^54,55^, if possible, or Efron’s method^56^, if not.

For the subgroup analysis of the time to the first occurrence of the main 5-component composite endpoint, the pre-specified Cox regression model also included an interaction between treatment group and the subgroup of interest, with the interaction *P* values evaluated using a score test.

#### Repeated measures analysis

An MMRM, which acknowledges the dependence/correlation among measures within a patient over time^57^, was used to analyze changes from baseline in eGFR and UACR. In this model, scheduled visits from weeks 4–208 (that is, including the additional laboratory data collection visits for European patients) for the full analysis set were included as fixed effects, and interactions with treatment and baseline allowed the treatment effect and the importance of the baseline to vary over visits. No structure was imposed on the dependence (covariance). Residual maximum likelihood (REML) was used for estimation, and, if convergence failed, then estimates of the covariance parameters were obtained from maximum likelihood estimation and used as starting values in REML. If the randomization value was missing, the screening value (if available) was used.

As was pre-specified, the estimated differences between treatment arms in the change from baseline at week 104 using the MMRM are presented. This timepoint was pre-specified to maximize the number observable while allowing sufficient duration of follow-up to evaluate the treatment effect reliably. Changes in UACR were analyzed as the average difference between the log-transformed baseline value and the log-transformed week 104 value, with the exponentiated difference between the differences in log-transformed values (baseline and week 104) for each treatment group. For ease of interpretation, these are expressed as relative percentage changes and relative percentage differences.

#### Annualized rate of change in eGFR

For the random effects eGFR slope analysis, a linear random regression model (linear random effects model) was used. Time was annualized and included as continuous along with the treatment effect (intercept) and an interaction between time and treatment (slope). To account for patient variation, patient effects and patient-by-time interaction were included as random (from a bivariate normal distributed with mean zero and an unstructured covariance matrix). The annual rate of change or slope was then compared between treatments, with the statistical significance given by that for the treatment-by-time interaction term^57^. As was pre-specified, eGFR slope was assessed during the acute (baseline to week 16), chronic (week 20 to end of trial) and total (baseline to end of trial) periods. Therefore, the acute slope included data only for the European patients for whom these timepoints were evaluated.

### Correlation and mediation analysis

We reported the correlation between the change in eGFR and change in body weight, systolic blood pressure and HbA_1c_. The correlations between the change in these factors and eGFR at week 104 were derived from an MMRM using the bivariate model as specified by Thiebaut et al.^58^. We estimated the percent mediation of the change in eGFR at week 104, potentially attributable to change in weight, by assessing how much the treatment effect on eGFR was altered by including the weight change in the regression model adjusted for baseline eGFR and baseline weight. From this model, the direct effect of semaglutide versus placebo on eGFR (hence, independent of change in body weight) was estimated. The total effect of semaglutide versus placebo was estimated using the same model but without change in body weight (the mediator) included. The percent mediated was calculated as the (total effect − direct effect) / total effect × 100%, and the standard error (and, hence, the 95% CI) of the percent mediated was estimated using the delta method. No intermediate observations between baseline and week 104 were used in the analysis^59^.

### Reporting summary

Further information on research design is available in the Nature Portfolio Reporting Summary linked to this article.

## Online content

Any methods, additional references, Nature Portfolio reporting summaries, source data, extended data, supplementary information, acknowledgements, peer review information; details of author contributions and competing interests; and statements of data and code availability are available at 10.1038/s41591-024-03015-5.

### Supplementary information

Reporting Summary

Supplementary Tables 1–5 Supplementary Table 1 Baseline demographics and clinical characteristics of the patients in the SELECT trial. Supplementary Table 2 Correlation of changes in eGFR at week 104 with changes in body weight, blood pressure and HbA1c. Supplementary Table 3 Effect of semaglutide 2.4 mg on time-to-event kidney endpoints. Supplementary Table 4 Adverse events by baseline eGFR. Supplementary Table 5 Complete list of kidney outcomes assessed.

## Data Availability

Data will be shared with bona fide researchers who submit a research proposal approved by the independent review board. Individual patient data will be shared in datasets in a de-identified and anonymized format. Data will be made available after research completion and approval of the product and product use in the European Union and the United States. Information about data access request proposals can be found at https://www.novonordisk-trials.com/.
